# Neutral detergent fiber rather than other dietary fiber types as an independent variable increases the accuracy of prediction equation for digestible energy in feeds for growing pigs

**DOI:** 10.5713/ajas.19.0103

**Published:** 2019-07-01

**Authors:** Hyunjun Choi, Jung Yeol Sung, Beob Gyun Kim

**Affiliations:** 1 Department of Animal Science and Technology, Konkuk University, Seoul 05029, Korea

**Keywords:** Digestible Energy, Neutral Detergent Fiber, Prediction Equation, Total Dietary Fiber

## Abstract

**Objective:**

The objectives were to investigate correlations between energy digestibility (digestible energy [DE]:gross energy [GE]) and various fiber types including crude fiber (CF), total dietary fiber (TDF), soluble dietary fiber (SDF), insoluble dietary fiber (IDF), neutral detergent fiber (NDF), and acid detergent fiber (ADF), and to develop prediction equations for estimating DE in feed ingredients and diets for growing pigs.

**Methods:**

A total of 289 data with DE values and chemical composition of feeds from 39 studies were used to develop prediction equations for DE. The equations were validated using values provided by the National Research Council.

**Results:**

The DE values in feed ingredients ranged from 2,011 to 4,590 kcal/kg dry matter (DM) and those in diets ranged from 2,801 to 4,203 kcal/kg DM. In feed ingredients, DE:GE was negatively correlated (p<0.001) with NDF (r = −0.84), IDF (r = −0.83), TDF (r = −0.82), ADF (r = −0.78), and CF (r = −0.72). A best-fitting model for DE (kcal/kg) in feed ingredients was: 1,356 + (0.704 × GE, kcal/kg) − (60.3 × ash, %) − (27.7 × NDF, %) with R^2^ = 0.80 and p<0.001. In diets, DE:GE was negatively correlated (p<0.01) with NDF (r = −0.72), IDF (r = −0.61), TDF (r = −0.52), CF (r = −0.45), and ADF (r = −0.34). A best-fitting model for DE (kcal/kg) in diets was: 1,551 + (0.606 × GE, kcal/kg) − (22.1 × ash, %) − (25.6 × NDF, %) with R^2^ = 0.62 and p<0.001. All variables are expressed as DM basis. The equation developed for DE in feed ingredients had greater accuracy than a published equation for DE.

**Conclusion:**

All fiber types are reasonably good independent variables for predicting DE of swine feeds. The best-fitting model for predicting DE of feeds employed neutral detergent fiber as an independent variable.

## INTRODUCTION

The energy supply to animals using feed ingredients accounts for the largest portion of total feed cost. To determine available energy concentrations in feed ingredients fed to pigs, *in vivo* experiments have been widely conducted. However, animal experiments to determine the energy values of feed ingredients are time-consuming and expensive. Therefore, alternative methods to determine energy values of feedstuffs have been developed. As one of the alternative methods, prediction equations have been developed to determine digestible energy (DE) values using the chemical composition of feed ingredients [1–4] and diets [5,6] for pigs.

In previously reported DE predicting equations for swine feeds, dietary fiber was used as a negative independent variable [1,2,4,6] as the dietary fiber is less digestible than starch, protein, and fat [6]. Several fiber analysis procedures are available including the crude fiber (CF) analysis [7], the detergent fiber procedure [8], and total dietary fiber (TDF) procedure [9]. Among the fiber analyzing procedures, the TDF procedure may provide an accurate estimate of fiber because TDF procedure takes the soluble dietary fiber (SDF) into account [10]. However, to our knowledge, there has been very limited effort to employ TDF as an independent variable for predicting DE in swine feeds. Therefore, the objectives of the present study were to investigate correlations between energy digestibility and various fiber types including CF, TDF, insoluble dietary fiber (IDF), SDF, neutral detergent fiber (NDF), and acid detergent fiber (ADF) and to develop and validate prediction equations for estimating DE using a fiber type as an independent variable for swine feeds.

## MATERIALS AND METHODS

### Data collection

A total of 289 data (105 feed ingredients and 184 diets) with DE values and chemical composition of feeds from 39 research papers were used to develop prediction equations for DE concentration. For the literature search in PubMed and Google Scholar, keywords used were DE, energy digestibility, nutrient digestibility, fiber, and pigs. The papers found were manually screened based on the title and the experimental procedures. During this screening process, data from nursery pigs or sows were removed. When TDF values for an ingredient is not available, the data were not used in the present work. The database consisted of crude protein (CP), ether extract (EE), ash, CF, NDF, ADF, TDF, IDF, SDF, and gross energy (GE) in the feeds (% or kcal/kg of DM basis). When an analyzed fiber concentration was not provided in the literature, the dietary fiber concentration was calculated based on the inclusion rate of feed ingredients and the fiber concentration of each ingredient (Table 1). Additionally, TDF concentration less than NDF concentration in feed ingredient was excluded from the database.

### Statistical analysis

Correlation coefficients among the chemical compositions (CP, EE, ash, CF, NDF, ADF, TDF, IDF, and SDF), energy digestibility coefficient (DE:GE), and energy concentrations (GE and DE) in feed ingredients and diets were determined by CORR procedure of SAS (SAS Inst. Inc., Cary, NC, USA). Prediction equations for DE in feed ingredients and diets were generated by PROC REG of SAS using GE, ash, NDF, IDF, TDF, and CF in feed ingredients and diets as independent variables. The statistical significance was determined as p<0.05. Redundant variables were excluded based on root mean square error (RMSE), R^2^, and p-values. The accuracy of prediction equations for DE in the present study and a previously published equation by Noblet and Perez [6] were assessed by regressing the determined DE values from feed ingredient composition of NRC [10] minus the calculated DE value on the each calculated value centered to the mean [11]. To validate prediction equation for DE, only data corresponding to the range of chemical compositions used for developing equations in the present study were employed.

## RESULTS

Most nutrient and energy concentrations in feed ingredients were more variable than those in diet based on coefficients of variation (Table 2). The NDF concentrations in feed ingredients ranged from 7.2% to 63.2% while those in diets ranged from 5.1% to 34.4% on DM basis. The DE values in feed ingredients ranged from 2,011 to 4,590 kcal/kg DM, and those in diets ranged from 2,801 to 4,203 kcal/kg DM.

Dietary fibers including CF, TDF, IDF, SDF, NDF, and ADF were positively correlated with each other in feed ingredients (r = 0.26 to 0.99; p<0.01; Table 3) and diets (r = 0.32 to 0.93; p<0.001; Table 4). The DE values in feed ingredients were positively correlated (p<0.001) with EE (r = 0.37), GE (r = 0.47), and DE:GE (r = 0.81; Table 3). The DE:GE of feed ingredients was negatively correlated (p<0.001) with NDF (r = −0.84), IDF (r = −0.83), TDF (r = −0.82), ADF (r = −0.78), and CF (r = −0.72). The best-fitting model for DE in feed ingredients was: DE (kcal/kg DM) = 1,356 + (0.704 × GE, kcal/kg DM) − (60.25 × ash, %) − (27.73 × NDF, %) with RMSE = 243, R^2^ = 0.80 and p<0.001 (Equation 1; Table 5). All nutrient variables are expressed as DM basis.

The DE values in diets were also positively correlated (p < 0.01) with EE (r = 0.21), GE (r = 0.47), and DE:GE (r = 0.67; Table 4). The DE:GE of diets was negatively correlated (p< 0.001) with NDF (r = −0.72), IDF (r = −0.61), TDF (r = −0.52), CF (r = −0.45), and ADF (r = −0.34). The best-fitting model for DE in diets was: DE (kcal/kg DM) = 1,551 + (0.606 × GE, kcal/kg DM) − (22.07 × ash, %) − (25.55 × NDF, %) with RMSE = 143, R^2^ = 0.62 and p<0.001 (Equation 5; Table 5). All nutrient variables are expressed as DM basis.

**Figure 1:**
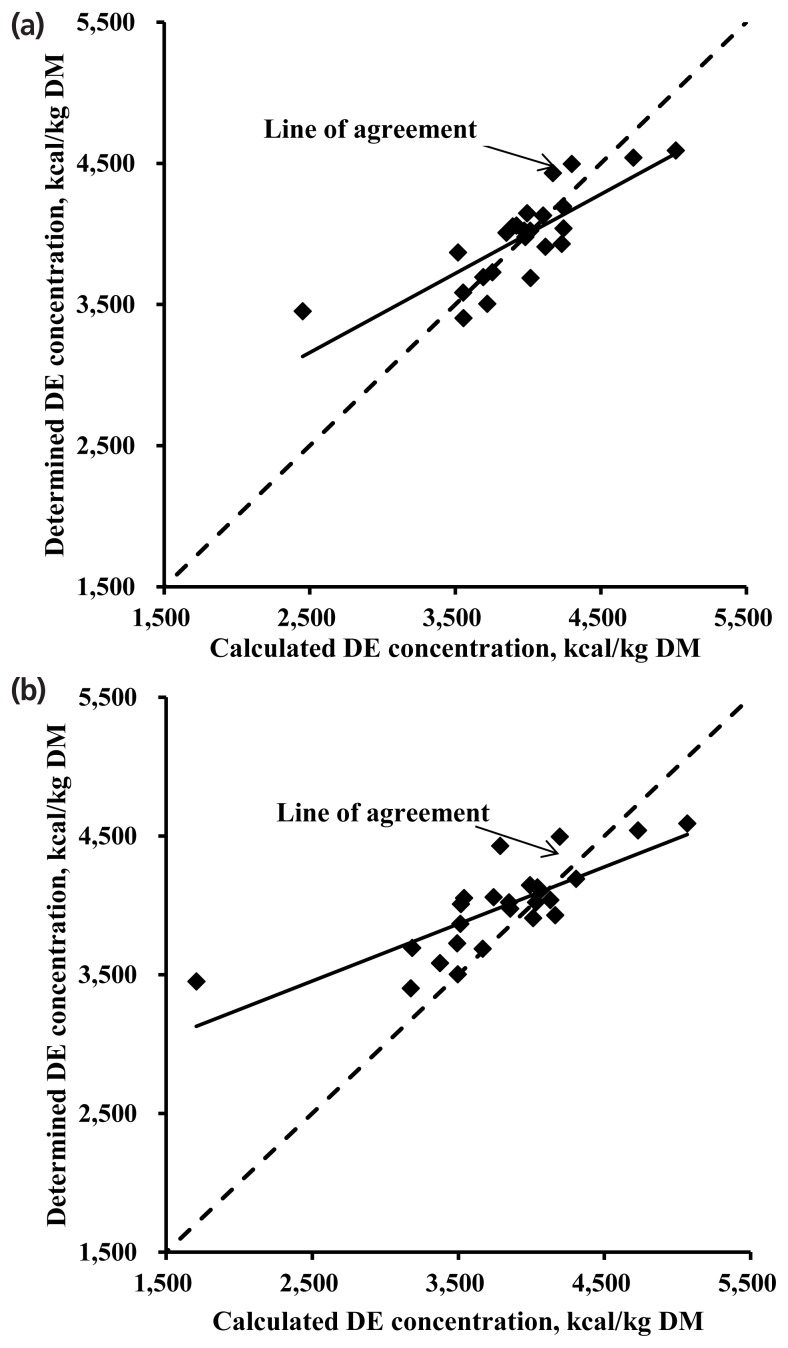
Comparison of determined and calculated digestible energy (DE, kcal/kg [DM]) using the determined DE value of feed ingredients from NRC [10] (n = 24). The prediction equation for DE in feed ingredient in the present study was: DE (kcal/kg DM) = 1,356 + 0.704 × gross energy (kcal/kg DM) − 60.25 × ash (% DM) − 27.73 × neutral detergent fiber (% DM) and published equation from Noblet and Perez [6] was: DE (kcal/kg DM) = 1,161 + 0.749 × gross energy (kcal/kg DM) − 4.3 × ash (% DM) − 4.1 × neutral detergent fiber (% DM). (a) For the regression analysis (determined – calculated DE vs calculated DE – average of calculated DE) using the equation in the present study, the intercept (18.2±41.3; p = 0.664) was not different from 0, whereas the slope (−0.439±0.089; p<0.001) was less than 0. (b) Using the model from Noblet and Perez [6], the intercept (205.3±43.0; p<0.001) and slope (−0.588±0.070; p<0.001) were different from 0.

The determined DE values of feed ingredients presented by the NRC [10] were plotted against a calculated DE values using an equation developed in the present work employing GE, NDF, and ash as independent variables and using an equation suggested by Noblet and Perez [6] (Figure 1). When the equation developed in the present work was tested using the NRC [10] data, the intercept representing a mean bias was not different from 0 but the slope representing a linear bias was different from 0 (p<0.001; Figure 1a). For the equation suggested by Noblet and Perez [6], both intercept and slope were different from 0 (p<0.001; Figure 1b) in the model validation results.

## DISCUSSION

The data of CF, NDF, and ADF used in the present study were in good agreement with NRC [10] and Sauvant et al [12]. When a TDF concentration was less than an NDF concentration in a feed ingredient, the data were not used for equation development because theoretically TDF includes SDF such as pectin, *β*-glucan, and gum [9,10].

When collecting data to develop an accurate prediction equation for DE, 2 factors were considered. First, only data of DE:GE were collected from pigs fed mash-form diets because feed processing may affect DE:GE [13]. Second, data derived from less than 20 kg of initial body weight of pigs were excluded. This is because the energy digestibility of feed ingredients [14] and diet [15] of nursery pigs would be less than that of growing and finishing pigs due to the immature digestive capacity and relatively small intestine size of nursery pigs [16].

Energy or nutrient digestibility is dependent on physico chemical characteristics of dietary fiber in feeds [17–19]. Even though TDF, IDF, and SDF are regarded as dietary fiber, the impact of each dietary fiber on digestibility differs. The energy digestibility coefficients were greater in growing pigs and sows fed high-SDF diets compared with pigs fed high-IDF diets [17,18]. In the same manner to *in vivo* studies, *in vitro* total tract disappearance of DM and organic matter had greater correlation with IDF than TDF [20]. These results indicate that the TDF, IDF, and SDF may differently affect energy digestibility due to the different physicochemical properties. Generally, IDF is less fermentable than SDF and the passage rate of digesta is most likely to be increased by IDF rather than SDF due to greater fecal bulk inducing intestinal motility and peristaltic wave in the gastrointestinal tract [21]. Also, the SDF is less-lignified than IDF [22] leading to increased digesta viscosity [23] and enzymatic digestion compared with IDF [24]. For these reasons, energy digestibility of a high-SDF diet is greater than that in a high-IDF diet. As the influence of IDF on digestibility is largely different from that of SDF, the TDF which is the sum of IDF and SDF would be less correlated with DE:GE compared with IDF in diets (r = −0.52 vs −0.61; Table 4). However, the DE:GE and DE were much more correlated with TDF than IDF in the work of Navarro et al [25]. The reason for this inconsistency may be the specific ingredient composition in the experiment by Navarro et al [25] who used synthetic cellulose and pectin to represent high-IDF and high-SDF source, respectively. In the present study, however, the data employing synthetic cellulose or pectin were not used.

The DE was calculated by multiplying GE concentration by DE:GE. In the present work, TDF and IDF were negatively correlated with DE:GE in feed ingredients whereas those fiber components were positively correlated with GE resulting in weakened negative correlation between those fiber components and DE (Table 3). These results are supported by a recent study [4]. For this reason, TDF may have shown less accuracy in predicting DE compared with NDF in feed ingredients. However, Anderson et al [26] and Kerr et al [2] reported that TDF had greater R^2^ than NDF to predict DE values of feed ingredients. The reason for this inconsistency may be due to the differences in analyzed TDF concentrations in corn-byproducts. In the present database, TDF concentrations in corn-byproducts such as distillers dried grains with solubles was greater than NDF concentration whereas TDF was less than NDF in Anderson et al [26] and Kerr et al [2]. Feed ingredients used to develop equations would be an important factor for the inconsistent results. In contrast to the previous studies [2,26], high-SDF feed ingredients such as barley and sugar beet pulp were used to develope equations in the present work.

The NDF and IDF concentrations in the same ingredients have a similar range except for a high-IDF ingredient (cellulose) and a high-SDF ingredient (pectin) in Navarro et al [25]. Although NDF and IDF values were comparable in most of feed ingredients or diets, NDF was the most accurate independent variable compared with other dietary fibers in the current study. This was unexpected because TDF more accurately represents the sum of fibers in a feed ingredient or diet compared with other dietary fibers including CF, SDF, IDF, NDF, and ADF. This result may be attributed to the analysis errors of the TDF procedure. The TDF procedure (TDF, IDF, and SDF) had less reproducibility and repeatability than the detergent fiber procedure [7]. Additionally, the IDF and SDF had different physicochemical characteristics which may decrease correlation between TDF and GE [20]. The characteristics of dietary fiber may contribute to the accuracy of DE estimation using TDF as an independent variable. In present work, however, NDF showed the greatest accuracy for estimating DE values perhaps because NDF had no significant correlation with GE. Therefore, further research is warranted to compare the detergent fiber procedure and TDF procedure as an independent variable on estimating DE values.

The best-fitting model for DE of feed ingredients in the present work had a better accuracy than an equation from Noblet and Perez [6] who used NDF as an independent variable (Figure 1). When developing a prediction equation, a wide range of chemical composition is desirable for high applicability [5]. The chemical compositions in the work by Noblet and Perez [6] had a relatively narrower range than those of the present work.

A limitation of the present work is that only DE-predicting equations are reported. The relationship between energy digestibility and fiber types was mainly addressed. When collecting data from the literature, quite a few experiments employed an index method and did not report metabolizable energy values. Further research is warranted to develop prediction models for metabolizable energy and net energy.

## CONCLUSION

The energy digestibility may be less affected by SDF than IDF. The GE is an important factor for predicting DE. The DE in swine feed ingredients and diets can be fairly accurately estimated using equations with NDF compared with other fiber types.

## Figures and Tables

**Table 1:** Nutrient composition of feed ingredients1) (as-fed basis)

Items	Analyzed composition (%)				

	n2)	DM	TDF	IDF	SDF
Alfalfa meal (lucerne hay)	1	94.60	59.31	56.76	2.46
Barley, dehulled	1	89.29	10.88	6.34	4.54
Barley, hulless	7	88.28	19.47	15.69	3.79
Canola expeller	2 (1)	94.96	28.10	26.75	3.75
Canola meal, *B. juncea* yellow	3	89.57	25.43	NA	NA
Canola meal, *B. napus* black	3 (2)	88.93	28.67	25.44	0.98
Copra expeller	1	96.54	43.84	42.05	1.79
Corn	15 (2)	86.73	11.94	10.53	1.28
Corn bran	2 (1)	86.25	42.18	37.19	4.76
Corn bran with solubles	3 (2)	93.82	24.89	23.90	1.40
Corn DDGS, 5 to 9% oil	7 (5)	88.32	33.64	35.12	1.01
Corn DDGS, 9%>oil	6 (3)	89.45	33.00	28.88	2.49
Corn germ meal	3	90.04	36.55	33.25	3.30
High-protein DDGS	1	86.50	34.20	31.80	2.40
Oats hulls	1	92.60	71.30	70.60	0.70
SBM, 44% CP	1	88.05	19.25	16.94	2.31
SBM, 46% CP	5 (3)	91.52	17.56	15.97	1.47
SBM, 47% CP	3	88.29	16.91	15.86	1.05
SBM, 48% CP	5	89.27	17.12	15.53	1.59
Sorghum	1	89.50	8.30	7.80	0.50
Soybean hulls	4	88.62	68.22	63.60	4.61
Sugar beet pulp	5	89.90	59.71	47.77	12.05
Wheat	5	88.59	12.18	10.79	1.38
Wheat bran	4	89.75	44.54	40.71	3.83
Wheat DDGS	1	89.30	31.20	30.60	0.50
Wheat middlings	15	88.57	37.00	34.61	2.44
Wheat millrun, 16.8% CP	1	88.80	38.00	35.50	2.42

DM, dry matter; TDF, total dietary fiber; IDF, insoluble dietary fiber; SDF, soluble dietary fiber; NA, not available; DDGS, distillers dried grains with solubles; SBM, soybean meal; CP, crude protein.

1) The DM, TDF, IDF, and SDF data were from 32 research papers.

2) The number of observations in the parenthesis represents the number of papers that reported only TDF but not IDF or SDF.

**Table 2:** Variability of digestibility coefficient, energy values, and nutrient composition of feed ingredients and diets1) (dry matter basis)

Items	Feed ingredient						Diet					
	
	n	Mean	SD	Min.	Max.	CV (%)	n	Mean	SD	Min.	Max.	CV (%)
Chemical composition (%)												
Crude protein	105	26.86	12.95	6.22	55.60	48.2	184	18.24	4.19	7.89	28.72	22.9
Ether extract	105	5.99	4.11	0.49	14.80	68.7	184	4.02	2.21	0.15	11.70	55.1
Ash	105	5.23	2.07	1.08	9.07	39.6	184	5.67	1.18	1.32	8.86	20.7
Crude fiber	105	8.78	5.06	1.26	43.44	57.7	184	4.64	2.36	1.94	19.80	50.9
Neutral detergent fiber	105	29.40	12.82	7.16	63.15	43.6	184	15.17	5.46	5.09	34.40	36.0
Acid detergent fiber	105	11.64	6.38	1.38	45.94	54.8	184	5.96	3.20	1.76	22.21	53.7
TDF	105	33.24	12.68	6.22	76.94	38.1	184	19.06	6.53	8.08	43.22	34.3
IDF	104	30.75	12.10	5.51	70.88	39.4	183	17.03	5.41	8.40	37.80	31.8
SDF	104	2.27	1.33	0.52	6.06	58.9	183	2.35	2.05	0.37	13.56	87.0
GE	105	4,765	467	3,523	5,634	9.8	184	4,402	196	3,859	4,939	4.5
DE:GE	105	0.750	0.100	0.469	0.959	13.3	184	0.842	0.048	0.651	0.980	5.7
DE	105	3,582	538	2,011	4,590	15.0	184	3,706	232	2,801	4,203	6.3

SD, standard deviation; CV, coefficient of variation; TDF, total dietary fiber; IDF, insoluble dietary fiber; SDF, soluble dietary fiber; GE, gross energy; DE, digestible energy.

1) The data were based on 39 research papers; TDF, IDF, and SDF were presented in 15 studies; when TDF, IDF, and SDF contents are not available, the fiber contents were calculated based on the ingredient composition and the fiber concentrations provided in Table 1 (n = 24).

**Table 3:** Correlation coefficients among chemical composition and digestible energy to gross energy and digestible energy of feed ingredients

Items	EE	Ash	CF	NDF	ADF	TDF	IDF	SDF	GE	DE:GE	DE
CP	0.16	0.58***	−0.07	−0.14	0.18	0.00	0.03	−0.32***	0.56***	−0.03	0.30**
EE	-	−0.04	0.06	0.27**	0.12	0.31**	0.30**	0.03	0.80***	−0.13	0.37***
Ash		-	0.34***	0.34***	0.49***	0.39***	0.40***	0.03	0.16	−0.48***	−0.36***
CF			-	0.81***	0.83***	0.86***	0.86***	0.53***	0.03	−0.72***	−0.62***
NDF				-	0.72***	0.95***	0.94***	0.53***	0.15	−0.84***	−0.65***
ADF					-	0.74***	0.74***	0.26**	0.18	−0.78***	−0.58***
TDF						-	0.99***	0.58***	0.25*	−0.82***	−0.57***
IDF							-	0.48***	0.26**	−0.83***	−0.57***
SDF								-	−0.15	−0.33***	−0.36***
GE									-	−0.11	0.47***
DE:GE										-	0.81***

EE, ether extract; CF, crude fiber; NDF, neutral detergent fiber; ADF, acid detergent fiber; TDF, total dietary fiber; IDF, insoluble dietary fiber; SDF, soluble dietary fiber; GE, gross energy; DE, digestible energy; CP, crude protein.

* p<0.05,

** p<0.01, and

*** p<0.001.

**Table 4:** Correlation coefficients between chemical composition and digestible energy to gross energy and digestible energy of diets

Items	EE	Ash	CF	NDF	ADF	TDF	IDF	SDF	GE	DE:GE	DE
CP	0.02	0.39***	0.08	0.16*	0.14	0.23**	0.24	−0.04	0.29***	−0.13	0.12
EE	-	−0.01	0.09	0.02	0.11	0.01	0.14	−0.18*	0.54***	−0.16*	0.21**
Ash		-	0.25***	0.21**	0.15*	0.24**	0.24**	0.14	−0.30***	−0.20**	−0.39***
CF			-	0.79***	0.82***	0.82***	0.82***	0.44***	−0.03	−0.45***	−0.46***
NDF				-	0.67***	0.87***	0.89***	0.39***	0.12	−0.72***	−0.56***
ADF					-	0.69***	0.66***	0.38***	0.06	−0.34***	−0.31***
TDF						-	0.93***	0.57***	0.07	−0.52***	−0.39***
IDF							-	0.32***	0.19	−0.61***	−0.40***
SDF								-	−0.28***	−0.10	−0.24**
GE									-	−0.25***	0.47***
DE:GE										-	0.67***

EE, ether extract; CF, crude fiber; NDF, neutral detergent fiber; ADF, acid detergent fiber; TDF, total dietary fiber; IDF, insoluble dietary fiber; SDF, soluble dietary fiber; GE, gross energy; DE, digestible energy; CP, crude protein.

* p<0.05,

** p<0.01, and

*** p<0.001.

**Table 5:** Prediction equations for digestible energy in feed ingredients and diets (kcal/kg DM basis)

Items	Regression coefficient parameter (% DM basis)							Statistical parameter		
	
	Intercept	GE (kcal/kg)	Ash	NDF	IDF	TDF	CF	RMSE	R^2^	p-value
Feed ingredient										
Equation 1	1,356	0.704	−60.25	−27.73	-	-	-	243	0.80	<0.001
SE	245	0.05	12.31	1.99	-	-	-	-	-	-
p-value	<0.001	<0.001	<0.001	<0.001	-	-	-	-	-	-
Equation 2	1,042	0.782	−51.32	-	−29.68	-	-	259	0.77	<0.001
SE	262	0.06	13.54	-	2.36	-	-	-	-	-
p-value	<0.001	<0.001	<0.001	-	<0.001	-	-	-	-	-
Equation 3	1,112	0.773	−55.36	-	-	−27.79	-	262	0.77	<0.001
SE	264	0.06	13.49	-	-	2.25	-	-	-	-
p-value	<0.001	<0.001	<0.001	-	-	<0.001	-	-	-	-
Equation 4	1,532	0.610	−67.42	-	-	-	−57.51	309	0.68	<0.001
SE	314	0.07	15.79	-	-	-	6.38	-	-	-
p-value	<0.001	<0.001	<0.001	-	-	-	<0.001	-	-	-
Diet										
Equation 5	1,551	0.606	−22.07	−25.55	-	-	-	143	0.62	<0.001
SE	273	0.06	9.76	2.02	-	-	-	-	-	-
p-value	<0.001	<0.001	0.025	<0.001	-	-	-	-	-	-
Equation 6	1,488	0.615	−25.78	-	−19.96	-	-	168	0.48	<0.001
SE	325	0.07	11.81	-	2.47	-	-	-	-	-
p-value	<0.001	<0.001	0.030	-	<0.001	-	-	-	-	-
Equation 7	1,936	0.486	−32.72	-	-	-	−39.62	174	0.44	<0.001
SE	329	0.07	11.86	-	-	-	5.64	-	-	-
p-value	<0.001	<0.001	0.006	-	-	-	<0.001	-	-	-
Equation 8	1,808	0.532	−32.71	-	-	−13.48	-	177	0.42	<0.001
SE	336	0.07	12.12	-	-	2.09	-	-	-	-
p-value	<0.001	<0.001	0.008	-	-	<0.001	-	-	-	-

DM, dry matter; GE, gross energy; NDF, neutral detergent fiber; IDF, insoluble dietary fiber; TDF, total dietary fiber; CF, crude fiber; RMSE, root mean square error; SE, standard error.
